# Prevention of diet-induced hepatic steatosis and hepatic insulin resistance by second generation antisense oligonucleotides targeted to the longevity gene *mIndy (Slc13a5)*

**DOI:** 10.18632/aging.100854

**Published:** 2015-12-05

**Authors:** Dominik H. Pesta, Rachel J. Perry, Fitsum Guebre-Egziabher, Dongyan Zhang, Michael Jurczak, Antje Fischer-Rosinsky, Martin A. Daniels, Diana M. Willmes, Sanjay Bhanot, Stefan R. Bornstein, Felix Knauf, Varman T. Samuel, Gerald I. Shulman, Andreas L. Birkenfeld

**Affiliations:** ^1^ Department of Internal Medicine, Yale University School of Medicine, New Haven, CT 06519, USA; ^2^ Cellular & Molecular Physiology, Yale University School of Medicine, New Haven, CT 06519, USA; ^3^ Howard Hughes Medical Institute, Yale University School of Medicine, New Haven, CT 06519, USA; ^4^ Department of Sport Science, Medical Section, University of Innsbruck, Innsbruck, Austria; ^5^ Department of Visceral, Transplant, and Thoracic Surgery, D. Swarovski Research Laboratory, Medical University of Innsbruck, Innsbruck, Austria; ^6^ Institute for Clinical Diabetology, German Diabetes Center, Leibniz Center for Diabetes Research at Heinrich-Heine University Düsseldorf, German Center for Diabetes Research, Partner Düsseldorf, Düsseldorf, Germany; ^7^ Charité – University School of Medicine, Department of Endocrinology, Diabetes and Nutrition, Berlin, Germany; ^8^ Section of Metabolic Vascular Medicine, Medical Clinic III and Paul Langerhans Institute Dresden (PLID), TU Dresden, Germany; ^9^ German Center for Diabetes Research (DZD), Dresden, Germany; ^10^ Isis Pharmaceuticals, Carlsbad, CA 92008, USA; ^11^ Section of Diabetes and Nutritional Sciences, Rayne Institute, King's College London, London, UK; ^12^ University Clinic Erlangen, Erlangen, Germany; ^13^ Veterans Affairs Medical Center, West Haven, CT 06516, USA

**Keywords:** Indy, aging, hepatic insulin resistance, type 2 diabetes, Slc13a5

## Abstract

Reducing the expression of the *Indy* (*I'm Not Dead Yet*) gene in lower organisms extends life span by mechanisms resembling caloric restriction. Similarly, deletion of the mammalian homolog, *mIndy* (*Slc13a5*), encoding for a plasma membrane tricarboxylate transporter, protects from aging- and diet-induced adiposity and insulin resistance in mice. The organ specific contribution to this phenotype is unknown. We examined the impact of selective inducible hepatic knockdown of *mIndy* on whole body lipid and glucose metabolism using 2′-O-methoxyethyl chimeric anti-sense oligonucleotides (ASOs) in high-fat fed rats. 4-week treatment with 2′-O-methoxyethyl chimeric ASO reduced *mIndy* mRNA expression by 91% (P<0.001) compared to control ASO. Besides similar body weights between both groups, *mIndy*-ASO treatment lead to a 74% reduction in fasting plasma insulin concentrations as well as a 35% reduction in plasma triglycerides. Moreover, hepatic triglyceride content was significantly reduced by the knockdown of *mIndy,* likely mediating a trend to decreased basal rates of endogenous glucose production as well as an increased suppression of hepatic glucose production by 25% during a hyperinsulinemic-euglycemic clamp. Together, these data suggest that inducible liver-selective reduction of *mIndy* in rats is able to ameliorate hepatic steatosis and insulin resistance, conditions occurring with high calorie diets and during aging.

## INTRODUCTION

Reducing the expression of the *Indy* (I'm Not Dead Yet) gene in lower organisms extends life span by mechanism resembling caloric restriction [1–3]. In *D. melanogaster*, mutating the Indy gene reduces body fat content, insulin-like proteins and reactive oxygen species production, extending life span [1, 4]. Interestingly,* Indy* mRNA is down-regulated by dietary restriction in normal flies and it was shown that Indy long-lived flies share several phenotypes with long-lived calorie restricted flies [2]. Similarly, in *C.elegans*, knock down of the Indy homolog *CeNAC2* extends life span [5], an effect mediated at least in part via AMPK/aak2 [6]. The mammalian Indy homolog encoded protein mINDY (NaCT) is part of the SLC13 protein family, consisting of Na-carboxylate and Na-sulfate cotransporters in vertebrates, invertebrates, plants, and bacteria [7]. mINDY mediates the co-transport of citrate, succinate, and several other dicarboxylates across the plasma membrane together with sodium in an electrogenic manner [8, 9]. The amino acid sequence of the N-terminal sodium and the carboxy-binding motif is highly conserved between many species, from bacterium to rat to human [10].

Our laboratory has demonstrated that deletion of the *mIndy* gene (*Slc13a5*) protects mice from aging and high fat diet-induced adiposity, insulin resistance and hepatic steatosis [9]. Moreover, we were able to show that in rats, glucagon is a transcriptional regulator of the *mIndy* gene, inducing *mIndy* expression via a CREB-dependent mechanism [11]. The highest mRNA expression levels of *mIndy* in mammals are observed in the liver [9], however, liver specific contributions to its beneficial effects have not been determined so far. *mIndy* has been suggested to be a target for the treatment of aging- and life style induced metabolic diseases [7, 10, 12], and it is, thus, of high interest to understand which tissues need to be targeted to achieve the full beneficial effect.

Here, our aim was to investigate the effect of inducible and liver specific knock down of *mIndy* via the use of 2′-O-methoxyethyl chimeric antisense oligonucleotides (ASOs). We used this approach to test whether or not targeting *mIndy* in adult, high-fat fed rats, is sufficient to prevent hepatic steatosis and hepatic insulin resistance. Moreover, we chose this intervention because it resembles a therapeutic approach.

## RESULTS

### Effect on fasting parameters and liver fat content

**Figure 1:**
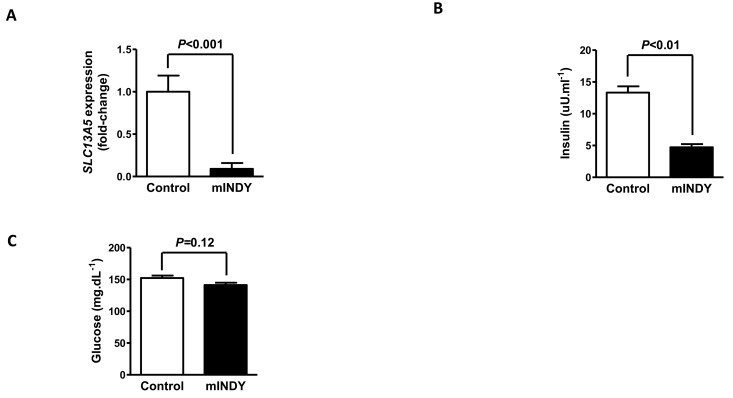
(**A**) After 4 weeks of 2′-O-methoxyethyl chimeric ASO treatment, *mIndy* mRNA expression was reduced by 91%. (**B**) Fasting plasma insulin concentrations were markedly reduced in the *mIndy* ASO treated rats. (**C**) Fasting glucose concentrations in the *mIndy* ASO and control ASO treated groups. All data are mean ± SEM, N=10 for each group; significances by double sided t-test.

After 4 weeks of 2′-O-methoxyethyl chimeric ASO treatment *mIndy* mRNA expression was reduced by 91% (P<0.001) in the treatment group (Fig. 1A). Body weight changes over the course of the study remained similar between the two groups.

The *mIndy* 2′-O-methoxyethyl chimeric ASO treated rats showed a 74% reduction in fasting plasma insulin (13.3 ± 1.0 μU.mL^−1^ vs. 4.7 ± 0.5 μU.mL^−1^, P<0.01) and their fasting plasma glucose concentration tended to be reduced (152.3 ± 3.8 mg.dL^−1^ for control group vs. 141.2 ± 3.7 mg.dL^−1^ for *mIndy* ASO treated, P=0.12) compared to the control group (Fig. 1B+C), suggesting improved insulin sensitivity in the *mIndy* ASO treated rats.

**Figure 2:**
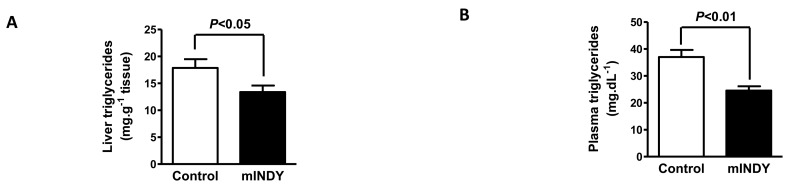
(**A**) 4 weeks of *mIndy* ASO treatment resulted in a 25% reduction in liver triglycerides and (**B**) a 35% reduction in plasma triglycerides. All data are mean ± SEM, N=10 for each group; significances by double sided t-test

Liver triglycerides in the *mIndy* ASO treated group were significantly reduced (17.9 ± 1.6 mg.g^−1^ tissue for control group vs. 13.4 ± 1.2 mg.g^−1^ tissue for *mIndy* ASO treated, P=0.04) (Fig. 2A). Consistent with this, we observed a significant 35% reduction of plasma triglycerides in the *mIndy* ASO treated group compared to the control group (37.0 ± 2.6 mg.dL^−1^ for control group vs. 24.6 ± 1.6 mg.dL^−1^ for *mIndy* ASO treated, P<0.01) (Fig. 2B). Other plasma metabolites were determined by an unbiased metabolomic approach using GC-TOF-MS. Profiles indicated significant changes in specific amino acids and total cholesterol and are listed in Table 1.

**Table 1:** 

Metabolite	Control (RPI)	mINDY ASO (RPI)	P – value
Glucose	5.95 ± 0.02	5.82 ± 0.01	0.012
Cholesterol	4.68 ± 0.02	4.58 ± 0.04	0.040
Palmitic Acid	4.66 ± 0.03	4.51 ± 0.05	0.022
Octadecanoic acid	4.28 ± 0.04	4.15 ± 0.04	0.025
Tryptophan	4.49 ± 0.02	4.39 ± 0.04	0.041
Glucopyranose	5.19 ± 0.03	5.07 ± 0.01	0.010
Tyrosine	4.52 ± 0.02	4.43 ± 0.04	0.035
Methionine	4.12 ± 0.01	4.04 ± 0.02	0.005
Ornithine	4.17 ± 0.02	4.08 ± 0.03	0.028

Plasma metabolite profiles as assessed by GC-TOF-MS metabolite profiling in *mIndy* ASO treated rats and animals from the control group. RPI=Relative peak intensities of the metabolites; normalized by the median of ^13^C-sorbitol intensities of all samples by the ^13^C-sorbitol intensity of the respective sample and log10 transformed. All data are mean ± SEM, N=10 for each group.

### Effect on insulin sensitivity

**Figure 3:**
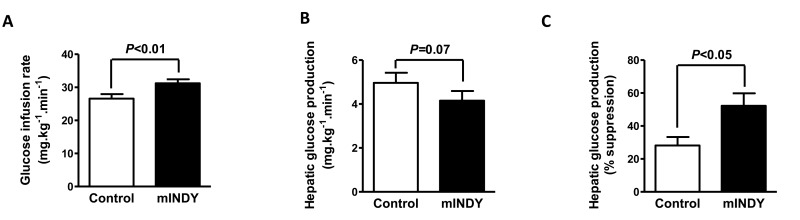
(**A**) Glucose infusion rate during the hyperinsulinemic-euglycemic clamp (HEC) is increased in *mIndy* ASO treated rats. (**B**) Trend for reduced hepatic glucose production during the HEC in the *mIndy* ASO treated rats. (**C**) Suppression of hepatic glucose production during the HEC was increased in *mIndy* ASO treated rats as compared to the control group. All data are mean ± SEM, N=10 for each group; significances by double sided t-test.

To determine the organ specific contribution to the improvement in glucose metabolism, the gold standard hyperinsulinemic-euglycemic clamp (HEC) test with stable isotope tracers was used. The glucose infusion rate during the clamp was significantly higher in *mIndy* ASO treated rats as compared to the control group (26.6 ± 1.4 mg.kg^−1^.min^−1^ for control group vs. 31.2 ± 1.2 mg.kg^−1^.min^−1^ for *mIndy* ASO treated rats, P<0.01) (Fig. 3A), confirming improved insulin sensitivity. The improvement was associated with a trend towards a reduction in basal rates of hepatic glucose production (5.0 ± 0.5 mg.kg^−1^.min^−1^ for control group vs. 4.2 ± 0.4 mg.kg^−1^.min^−1^ for *mIndy* ASO treated rats, P=0.07) during the HEC (Fig. 3B). Furthermore, hepatic insulin responsiveness was increased in the *mIndy* 2′-O-methoxyethyl chimeric ASO rats as reflected by increased suppression of hepatic glucose production during the HEC (28 ± 5% for control group vs. 52 ± 8% for *mIndy* ASO treated rats, P<0.05) (Fig. 3C).

## DISCUSSION

Our data are the first to show that inducible liver-specific knockdown of the mammalian homolog of the longevity gene *Indy* (Slc13a5) using second generation ASOs in rats ameliorates diet-induced hepatic steatosis, reduces plasma insulin, lipid and amino acid levels and improves hepatic insulin sensitivity independent of body weight. These features are core components of the metabolic syndrome that develops with high calorie diets [16] and aging [17, 18]. *mIndy* encodes for a citrate transporter located on the plasma membrane of hepatocytes [19, 20]. Citrate is a central metabolite associated with metabolic regulation [21] and aging [22]. Cytosolic citrate is converted to acetyl-coenzyme A (acetyl-CoA), the essential precursor for fatty acid, triglyceride and cholesterol biosynthesis. In our data set, we observed that reducing *mIndy* in the liver reduced hepatic as well as plasma triglyceride content. It is well established that reducing ectopic lipid deposition results in improved insulin sensitivity [16, 23], as shown in our study by marked reductions in basal insulin levels, a trend to improved basal hepatic glucose output and improved insulin mediated suppression of hepatic glucose production. In line with our data, reducing the conversion of cytosolic citrate to acetyl-CoA prevents liver lipid accumulation and insulin resistance [24] in mice. Moreover, we have shown that whole body deletion of *mIndy* reduces citrate uptake into the liver and prevents aging- and diet-induced accumulation of hepatic triglycerides and diacylglycerols , a mechanism well known to protect from hepatic insulin resistance [9, 16, 25, 26]. Reducing *mIndy* in primary human hepatocytes reduces hepatocellular lipid content [27] while overexpression or stimulation of *mIndy* in primary hepatocytes and cell lines increases hepatocellular citrate uptake and citrate derived lipid content [11, 28, 29].

Previous data indicated that reducing *Indy/mIndy* in lower organisms and mice lead to cellular and molecular processes that mediate a healthy aging process and longevity [1], i.e. increased mitochondrial biogenesis [3, 4, 9], reduced rate of reactive oxygen species production per mitochondrion [3], reduced body fat content [2, 5, 6, 9], increased PGC-1α expression [3, 4] and activated AMPK [6, 9]. Our data presented here add novel aspects. The insulin/igf1 pathway has been shown to be involved in mammalian regulation of lifespan and tumor growth [18, 30]. Moreover, restriction of specific amino acids, such as methionine [31], has been shown to extend lifespan in different species [32, 33]. Interestingly, our data indicate that selective hepatic reduction of *mIndy* markedly reduces fasting insulin levels, and our unbiased metabolomic data show that specific amino acid levels, i.e. methionine, tyrosin and ornithine, are reduced. Decreased levels of circulating amino acids have also been put into context of improved insulin sensitivity [34]. In humans, key serum amino acids levels change with aging [35]. Reduced amino acid concentrations might therefore resemble a phenotype going along with a healthy aging process. Cross-sectional and prospective analyses of large patient cohorts revealed that elevated plasma amino acid levels, especially of branched chain amino acids, tyrosine, alanine and phenylalanine, may contribute to an increased incidence of diabetes [36] and are also related to life span [37].

*mIndy* ASO treated rats further show reduced total cholesterol as well as stearic acid and palmitic acid levels in the metabolome profile. It is well established that serum free fatty acids (FFAs) are important contributing factors for the development of insulin resistance [16]. Along these lines, increased *de novo* lipid synthesis occurs via insulin-stimulated elongation of palmitic acid, contributing to increased stearic acid levels [38]. In humans, circulating levels of palmitic acid and stearic acid are correlated to insulin resistance while reduced levels of FFAs improve insulin resistance [38].

In contrast to whole body *mIndy*^−/−^ mice [9], *mIndy* ASO treatment did not affect body weight. Differences between the two models include a shorter timeframe of *mIndy* knockdown in the study presented here, the fact that knockdown is less effective than deletion and that other organs besides the liver may contribute to the body weight effect. In this context, it is important to note that mutations in the human *SLC13A5* gene have been reported in neonatal childhood epilepsy with teeth hypoplasia or hypodontia in a recent study [39], a feature not observed in the whole body *Slc13a5* knockout mice so far. Whether or not these mutations are causative is not entirely clear. From our data presented here, we can now conclude that selective hepatic reduction of *mIndy* is able to ameliorate hepatic steatosis and to improve diet-induced insulin resistance. Neuronal contribution seems not to be needed. Our data suggest that hepatic *mIndy* is an interesting target for the treatment of NAFLD and type 2 diabetes and first selective mINDY (SLC13A5) inhibitors have shown promise in this regard (40)Further studies need to address the important question whether or not the knockdown of *mIndy* in mammals will also promote longevity.

## METHODS

### Animals

All protocols were approved by the Yale University School of Medicine Animal Care and Use Committee. Male Sprague-Dawley rats were purchased from Charles River Laboratories at about 400 g. After the rats acclimated for at least one week, rats received i.p. injections over a period of 4 weeks of either 2′-O-methoxyethyl chimeric anti-sense oligonucleotides targeted against *mIndy* or a control ASO targeted against a sequence that does not match any known transcript in the rat. During the 4 weeks of ASO treatment, animals were fed a 60% high-fat diet based on safflower oil (Dyets, Bethlehem, Pennsylvania, USA) and body weights were monitored biweekly. All animals had *ad libitum* access to 6% sucrose water. After the treatment, rats underwent surgery under isoflurane anesthesia and catheters were inserted in the jugular vein and internal carotid artery. All animals were allowed to recover for at least 1 week before any further experiments were performed.

### Measurement of liver and plasma triglycerides

Liver triacylglycerides were extracted by the method of Bligh and Dyer [13] and measured spectrophotometrically with a commercial triglyceride reagent (Diagnostic Chemicals Limited [DCL], Oxford, Connecticut). Plasma was collected from the jugular vein of overnight fasted rats and assayed for plasma triglyceride concentrations using a commercial DCL triglyceride reagent.

### Hyperinsulinemic-euglycemic clamp

For the hyper-insulinemic-euglycemic clamp, after an overnight fast, awake, unrestrained rats first underwent a primed-continuous basal infusion of [6,6-^2^H_2_] glucose (5 mg.kg^−1^.min^−1^ prime for 5 min, 1 mg.kg^−1^.min^−1^ continuous infusion) through a catheter in the carotid artery. Blood samples (300 μl whole blood) were taken from a catheter in the jugular vein after 100, 110, and 120 min of infusion. This was followed by a HEC with regular insulin (40 mU.kg^−1^ bolus, 4 mU.kg^−1^.min^−1^) continuous infusion). Blood samples were taken at 15, 30, 45, 60, 75, 90, 100, 110, and 120 min of the clamp and glucose measured using a YSI Glucose Analyzer. Variable [6,6-^2^H_2_] glucose was given to maintain euglycemia (100-110 mg.dL^−1^) during the clamp. Glucose uptake in skeletal muscle was assessed after administration of a 40 μCi bolus of [^14^C]2-deoxyglucose at the end of the clamp and measured as described earlier [14].

### mRNA quantification by Real-Time PCR

Liver total RNA was isolated using the RNeasy kit per manufacturer's instructions (Qiagen, Valencia, California, USA), and qPCR was performed as described earlier [15]. Actin was used as a housekeeping gene.

### Plasma metabolite extraction, measurement, alignment and normalization

30 μl of the murine plasma was extracted with ice cold (−20°C) 400 μl 100% Methanol (^13^C-sorbitol added as an internal standard to control for technical variation). After shaking and centrifugation, the supernatant was vacuum-dried. GC-TOF-MS metabolite profiling was performed on a Leco Pegasus 3 time-of-flight mass spectrometer (Leco, St.Joseph, MI, USA). The Direct Thermal Desorption injector (ATAS GL International, The Netherlands) was coupled to an HP 5890 gas chromatograph and an autosampler with automatic derivatisation and liner exchange. This eliminates the impact of potential degradation or synthesis artifacts and sample carryover. During the derivatisation a retention time index standard mixture was also added. For detailed information refer to [9]. Chromatogram acquisition parameters were those described previously [9]. The results were exported from Leco Chroma TOF software (version 3.25) as cdf-files. Peak detection, retention time alignment, and library matching were performed with the R-script “Target Search”. Relative peak intensities of the metabolites were normalized by the median of ^13^C-sorbitol intensities of all samples by the ^13^C-sorbitol intensity of the respective sample and log10 transformed.

### Statistical analysis

All results are presented as mean ± SEM. Group comparisons (N=10 for each group) were performed by the 2-tailed unpaired Student's t-test or ANOVA where appropriate using Prism 6 for Windows software (GraphPad, Inc., La Jolla, CA, USA). A P-value of 0.05 or less was considered statistically significant.

## Abbreviations

ASO: Anti-sense oligonucleotide
HEC: hyperinsulinemic-euglycemic clamp
NAFLD: nonalcoholic fatty liver disease
